# Multiple mechanisms underlie defective recognition of melanoma cells cultured in three-dimensional architectures by antigen-specific cytotoxic T lymphocytes

**DOI:** 10.1038/sj.bjc.6603664

**Published:** 2007-03-06

**Authors:** C Feder-Mengus, S Ghosh, W P Weber, S Wyler, P Zajac, L Terracciano, D Oertli, M Heberer, I Martin, G C Spagnoli, A Reschner

**Affiliations:** 1 ICFS, Departments of Surgery and Research, Basel University Hospital, Hebelstrasse 20, CH-4031 Basel, Switzerland; 2 Department of Pathology, Basel University Hospital, Hebelstrasse 20, CH-4031 Basel, Switzerland

**Keywords:** melanoma, immunorecognition, CTL, spheroids, antitumour response

## Abstract

Cancer cells’ growth in three-dimensional (3D) architectures promotes resistance to drugs, cytokines, or irradiation. We investigated effects of 3D culture as compared to monolayers (2D) on melanoma cells’ recognition by tumour-associated antigen (TAA)-specific HLA-A^*^0201-restricted cytotoxic T-lymphocytes (CTL). Culture of HBL, D10 (both HLA-A^*^0201+, TAA+) and NA8 (HLA-A^*^0201+, TAA−) melanoma cells on polyHEMA-coated plates, resulted in generation of 3D multicellular tumour spheroids (MCTS). Interferon-gamma (IFN-*γ*) production by HLA-A^*^0201-restricted Melan-A/MART-1_27–35_ or gp100_280–288_-specific CTL clones served as immunorecognition marker. Co-culture with melanoma MCTS, resulted in defective TAA recognition by CTL as compared to 2D as witnessed by decreased IFN-*γ* production and decreased Fas Ligand, perforin and granzyme B gene expression. A multiplicity of mechanisms were potentially involved. First, MCTS *per se* limit CTL capacity of recognising HLA class I restricted antigens by reducing exposed cell surfaces. Second, expression of melanoma differentiation antigens is downregulated in MCTS. Third, expression of HLA class I molecules can be downregulated in melanoma MCTS, possibly due to decreased interferon-regulating factor-1 gene expression. Fourth, lactic acid production is increased in MCTS, as compared to 2D. These data suggest that melanoma cells growing in 3D, even in the absence of immune selection, feature characteristics capable of dramatically inhibiting TAA recognition by specific CTL.

The identification of a large number of tumour-associated antigens (TAA) (Renkvist *et al*, 2001) has raised hopes of taking advantage of the enormous increase of knowledge stemming from basic immunology research to ameliorate the prognosis of neoplastic diseases by active antigen-specific immunotherapy, that is, by vaccination. Trials based on diverse immunisation procedures have been performed in different types of cancer and promising data have been reported (Nestle *et al*, 1998; Rosenberg *et al*, 1998; Thurner *et al*, 1999; Bedrosian *et al*, 2003; Slingluff *et al*, 2003).

Indeed, immune responses specific for TAA can be generated relatively easily ‘*in vitro*’ and ‘*in vivo*’, as detectable by phenotypic and functional assays. However, only in a minority of patients showing evidence of successful immunisation, clinical responses are also detectable.

Interestingly, experimental murine models indicate that tumour cells in suspension, regardless of their numbers, are frequently unable to produce life-threatening cancer outgrowth, as opposed to solid tumour fragments (Ochsenbein *et al*, 2001), while inducing specific immune responses. Thus, proliferation in structured architectures appears to represent a pre-requisite for cancer development.

In the human experimental setting, cytotoxicity assays or the functional monitoring of clinical immunotherapy trials are usually performed by utilising, as targets, cell lines, frequently of lympho/myeloid origin, expressing appropriate HLA alleles upon pulsing with specific peptides. Tumour-associated antigens recognition may eventually be confirmed by using, as additional targets, tumour cell lines expressing specific HLA determinants and transcribing relevant TAA-encoding genes. Current protocols typically imply the admixture of effector and target cells pelleted together in culture wells. The lack of correlation between data obtained ‘*in vitro*’ with these technologies and clinical data suggests that this model might not adequately account for critical aspects of the interaction between immunocompetent cells and cancers.

Three-dimensional (3D) culture models have been developed in the past decade, aiming at exploring radio or chemoresistance of tumour cells in ‘*in vitro*’ assays more closely related to ‘*in vivo*’ conditions than standard monolayers (Sutherland, 1988). In particular, multicellular tumour spheroids (MTCS) have been suggested to represent accurately early events of avascular tumour growth (Desoize and Jardillier, 2000).

Multicellular tumour spheroids remind *in vivo* cancers in their capacity to develop necrotic areas far from nutrient and oxygen supplies. Furthermore, cells cultured in MCTS are also similar to solid tumours in their proliferation dynamics (Gorlach *et al*, 1994), since at difference with monolayer cultures, they fit the Gompertz equation, classically used to quantitatively evaluate neoplastic growth (Chignola *et al*, 2000).

Three-dimensional growth of tumour cells *in vitro* has been shown to affect antigen recognition by specific cytotoxic T-lymphocytes (CTL). For instance, tumour infiltrating lymphocytes capable of killing autologous bladder tumour cells in 2D, failed to recognise targets when cultured in 3D (Dangles *et al*, 2002). Similarly, a CTL clone specific for a mutated *α*-actinin-4 peptide expressed by autologous lung cancer cells recognised poorly targets growing in MCTS, possibly due to a downregulation of HSP70 expression (Dangles-Marie *et al*, 2003).

Regarding melanoma, a cancer frequently targeted in clinical immunotherapy trials, we recently showed that the architecture of tumour cell growth determines specific gene expression profiles, of potentially high functional significance. Furthermore, we showed that the recognition of a melanoma TAA by a specific CTL clone was impaired when target cells were cultured in MCTS (Ghosh *et al*, 2005a, 2005b).

These data suggest that antigen-specific functional activities of CTL might be significantly altered in the presence of tumour cells growing in multilayered architectures. Underlying mechanisms, however, are still poorly investigated.

In this work, we explored the molecular bases of impaired recognition by CTL specific for TAA of melanoma cells cultured in three-dimensional architectures. We report that a multiplicity of mechanisms ranging from structural hindrances to downregulation of antigen and HLA expression to lactic acid overproduction concurrently limit the susceptibility of melanoma cells cultured in MCTS to the attack by TAA-specific CTL.

## MATERIALS AND METHODS

### Cell cultures

NA8 (courtesy of Dr Jotereau, Nantes, France), HBL and D10 cell lines (courtesy of Dr A Eberle, Basel, Switzerland) derive from metastatic melanoma and have widely been used in tumour immunology studies in the recent past (Gervois *et al*, 1996; Oertli *et al*, 2002; Zajac *et al*, 2003). They are all HLA-A^*^0201+. However, whereas HBL and D10 express typical melanoma differentiation TAA (Certa *et al*, 2001; Renkvist *et al*, 2001), NA8 does not (Certa *et al*, 2001).

Cell lines were routinely passaged in conventional 2D cultures in RPMI 1640 supplemented with 10 mM HEPES buffer, 1 mM sodium pyruvate, 2 mM non-essential amino acids, 2 mM glutamine, 100 *μ*g ml^−1^ Kanamycin (Invitrogen, Carlsbad, CA, USA) and 10% heat-inactivated FCS, hereafter referred to as complete medium.

Multicellular tumour spheroids were prepared in U-bottom 96-well plates previously coated with 50 *μ*g ml^−1^ poly-2-hydroxyethylmethacrylate (polyHEMA, Sigma, St Louis, MO, USA) solution, preventing cell binding as described (Folkman and Moscona, 1978). Cells proliferation was measured by the alamarBlue™ Assay (Serotec, Oxford, UK) (Hamid *et al*, 2004).

Cytotoxic T-lymphocyte clones were generated from peripheral blood CD8+ T cells of patients undergoing active antigen specific immunotherapy in the context of specific clinical trials, as described previously (Oertli *et al*, 2002; Zajac *et al*, 2003). Briefly, cells from bulk cultures showing evidence of antigen-specific cytotoxic activity, as detectable by ^51^Cr release assays, were cloned in 60-well Terasaki plates (Nunc, Glostrup, Denmark) at 0.3 cells per well in 20 *μ*l volumes in the presence of 10 000 irradiated allogenic PBMC/well, in RPMI 1640 supplemented with 10 mM HEPES buffer, 1 mM sodium pyruvate, 2 mM non-essential amino acids, 2 mM glutamine, 100 *μ*g ml^−1^ Kanamycin (Invitrogen, Carlsbad, CA, USA) and 5% pooled human serum (RPMI-HS), to which rIL-2 (200 U ml^−1^) and purified phytohemagglutinin (PHA, 0.5 *μ*g ml^−1^, Remel, Dartford, UK) were added. After 14 days, wells where cell growth was microscopically detectable were expanded in RPMI-HS supplemented with 100 U ml^−1^ rIL-2 and screened for antigen-specific cytotoxic activity by ^51^Cr release assay. Cytotoxic T-lymphocyte clones were maintained in RPMI-HS supplemented with 100 U ml^−1^ rIL-2 and restimulated periodically with PHA in the presence of irradiated allogenic PBMC. All assays reported here were performed at least 1 week after re-stimulation.

### Confocal microscopy

Cells were stained with PKH26 red fluorescent cell linker (Sigma-Aldrich, St Louis, MO, USA) following suppliers instructions, and cultured on polyHEMA-coated plates for 3 days to form 30 000 cells MCTS. Total CD8+ T cells from healthy donors and CTL clones specific for HLA-A^*^0201-restricted Melan-A/MART-1_27–35_ epitope were stained with CFSE (molecular Probes, Eugene, OR, USA). Cells (E : T ratio=2.5 : 1) were added to each MCTS and co-cultured for 1 day. Confocal images for the evaluation of total CD8+ T cells or CTL infiltration in NA8 and HBL MCTS, respectively, were obtained using a laser-scanning confocal microscope Zeiss LSM 510 (Carl Zeiss Microimaging Inc., Thornwood, NY, USA).

### Antigen recognition assays

Interferon-gamma production was used as antigen recognition assay. As target/stimulator cells, we used melanoma cells cultured in 2D or in MCTS. Cytotoxic T-lymphocyte specific for Melan-A/MART-1 or gp100 and used as effectors, were co-cultured with target cells at 2.5 : 1 ratio for 24 h. When indicated, assays were performed by using, as targets, HBL cells previously cultured in monolayers in the presence of 0–20 mM lactic acid (Sigma-Aldrich, St Louis, MO, USA) for 3 days. In all cases, supernatants were collected and IFN-*γ* secretion was measured by using human IFN-*γ* BD OptEIA™ ELISA Set (BD Biosciences, Franklin Lakes, NJ, USA). All samples were measured in triplicates.

### Quantification of gene expression by quantitative real-time PCR

Cells were collected at the indicated time points and washed in PBS. Total RNA was extracted using the RNeasy® Mini Kit protocol (Qiagen, Basel, Switzerland), treated by Deoxyribonuclease I (DNase I) (Invitrogen, Carlsbad, CA, USA), and reverse transcribed by using the Moloney Murine Leukemia Virus Reverse Transcriptase (M-MLV RT, Invitrogen, Carlsbad, CA, USA). Quantitative real-time PCR were performed in the ABI prism™ 7700 sequence detection system, using the TaqMan® Universal PCR Master Mix, No AmpErase® UNG (both from Applied Biosystems, Foster City, CA, USA).

Specific gene expression was quantitated by using the 2^−ΔΔ*C*_T_^, method (Winer *et al*, 1999; Livak and Schmittgen, 2001; Malec *et al*, 2004; Nieman *et al*, 2004). Normalisation of gene expression was performed using GAPDH as reference gene and data were expressed as ratio to reference samples.

### Primers and probes

Oncostatin M (OSM), interferon-regulating factor-1 (IRF-1), and microphtalmia-associated transcription factor (MITF) primers and probes are from pre-developed assays (Assays-on-Demand, Gene Expression Products (Applied Biosystems, Foster City, CA, USA)). Oligonucleotide primers and probes for c-*myc*, Melan-A/MART-1, gp100, and tyrosinase were generated using appropriate software (Primer Express™, Applied Biosystems, Foster City, CA, USA) from sequences obtained from the NCBI gene bank.

Furthermore, a number of sequences for primers and probes were derived from existing literature, as indicated below.


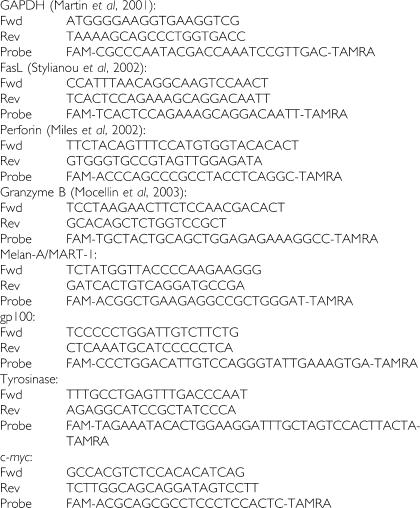


### Flow cytometry analysis

HLA class I expression was quantified using FITC-conjugated mAb specific for HLA-A^*^0201 or a similar reagent specific for a non-polymorphic determinant of HLA class I heavy chain (PharMingen, San Diego, CA, USA). The HBL, D10 or NA8 cells cultured in 2D were collected using Trypsin-EDTA (Invitrogen, Carlsbad, CA, USA) after 3 days culture. Accordingly, MCTS obtained after 3 days culture were disrupted by 5 min trypsinisation at 37°C. Cells were then incubated with specific or control mAbs, for 45 min at 4°C in the dark, washed twice in cold PBS, fixed 1 min in paraformaldehyde 1%, re-suspended in 200 *μ*l PBS, and analysed on a FACSCalibur® cytometer (Becton Dickinson, Franklin Lakes, NJ, USA).

### Lactic acid measurement

Quantification of lactic acid production by melanoma cells was performed after 3-day cultures in 2D or in 3D (30 000 cell density), using as immobilised enzyme biosensor, YSI 2300 STAT Glucose and Lactate analyser (YSI, Yellow Springs, OH, USA), following the suppliers' protocols.

### Statistical analysis

Statistical analysis software SPSS (Version 14.0, SPSS Inc., Chicago, IL, USA) was used for statistical analyses. Mann–Whitney *U*-tests were used to calculate asymptotic significance between two independent samples. All reported *P*-values were two-tailed and were considered to be statistically significant at *P*⩽0.05.

## RESULTS

### Morphological characterisation and growth pattern of melanoma spheroids

**Figure 1:**
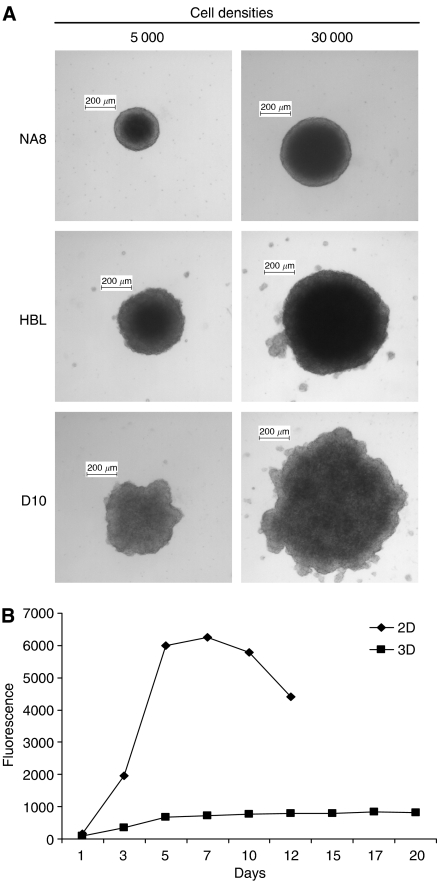
Generation and characterisation of MCTS of melanoma cells. (**A**) In all, 5000 or 30 000 melanoma cells (NA8, HBL, and D10) were cultured on polyHEMA-coated plates to prevent cell attachment for 3 days, resulting in the formation of MTCS of an average diameter of 300–800 *μ*m. (**B**) D10 cells proliferation was evaluated by alamarBlue™ Assay (Serotec, Oxford, UK). Divergent kinetics were detectable for D10 cells cultured in monolayer (2D) or MCTS (3D). Similar results were observed for NA8 (Ghosh *et al*, 2005b) and HBL cells (Ghosh *et al*, 2005a).

Melanoma cells were routinely maintained in monolayer (2D) cultures in complete medium. Upon culture on 96-well plates coated with polyHEMA preventing cell attachment (Folkman and Moscona, 1978), they formed 3D aggregates. Multicellular tumour spheroids consisting of 5000 or 30 000 cells showed 0.3–0.8 mm diameter (Figure 1A).

Consistent with previous results by our group (Ghosh *et al*, 2005a, 2005b), proliferation kinetics of cells cultured in 2D or 3D were dramatically different. Data reported in Figure 1B regarding D10 melanoma cell line as representative example indicate that proliferation in 2D cultures reached a plateau within 5 days, whereas, in contrast, no major modifications in cell numbers were detectable in MCTS within 20 days of culture.

### Morphology of the interaction between TAA-specific CTL and melanoma cells cultured in spheroids

First, we investigated the morphology of the interaction between melanoma cells cultured in 3D and immunocompetent cells, focusing on immature Dendritic Cells (iDC) capable of antigen uptake and processing and, most importantly, on CD8+ T cells, largely responsible for tumour-specific cytotoxic activities.

**Figure 2:**
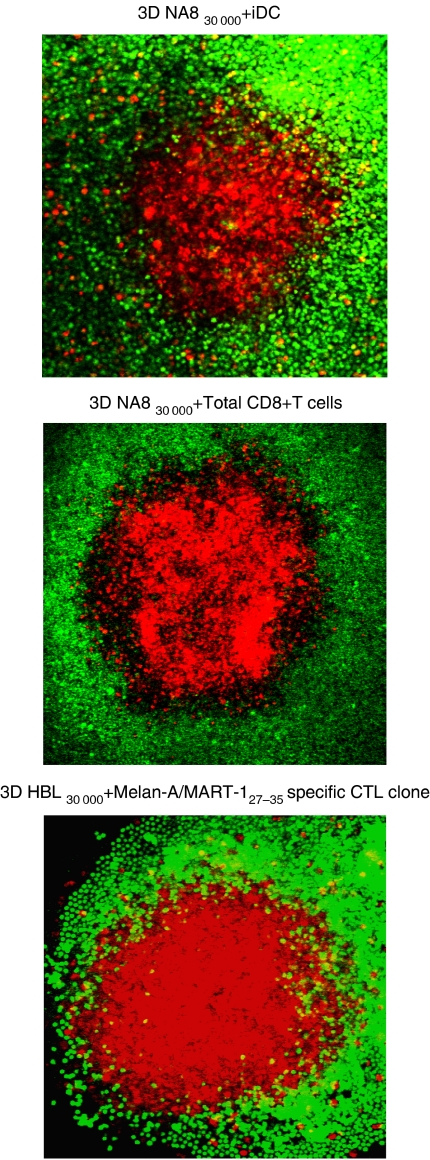
‘Non-brisk’ infiltration of melanoma MCTS by iDC, total CD8+ T cells and antigen-specific CTL. NA8 and HBL cells were stained with PKH26 red fluorochrome and cultured on polyHEMA-coated plates for 3 days to form 30 000 cells MCTS. Immature DC, total CD8+ T cells from a healthy donor and a CTL clone specific for HLA-A^*^0201-restricted Melan-A/MART-1_27–35_ epitope were labelled with CFSE. Cells were added at a 2.5 : 1 ratio to each MCTS and co-cultured for 24 h. Immature DC, total CD8+ T cells and TAA-specific CTL infiltration in NA8 and HBL MCTS, respectively, were analysed by confocal microscopy.

NA8 (HLA-A^*^0201+, Melan-A/MART-1-) or HBL (HLA-A^*^0201+, Melan-A/MART-1+) cells cultured as MCTS, iDC, total CD8+ T cells, and HLA-A^*^0201-restricted Melan-A/MART-1_27–35_-specific CTL clones were labelled with different fluorochromes. NA8 cells were co-cultured with either iDC or total CD8+ T cells. Furthermore, HBL cells were co-incubated for 24 h together with TAA-specific CTL clones. Confocal microscopy was then used to verify the consequences of this interaction at morphological level. Typically, iDC, CD8+ T cells in general and TAA-specific CTL in particular were unable to penetrate in deep the 3D architecture of MCTS, but rather tended to remain on their surfaces (Figure 2). These pictures closely reminded the ‘non-brisk’ infiltration of melanoma by T cells, as frequently observed in clinical tumour specimens (Mihm Jr *et al*, 1996; Bernsen *et al*, 2004).

### CTL clones display a differential capacity of recognising endogenously processed TAA in melanoma cells cultured in monolayers or in MCTS

Lack of MCTS infiltration by TAA-specific CTL hinted at a possible defective killing of tumour cells cultured in spheroids. Indeed, growth in 3D architectures was suggested previously to prevent the recognition of TAA by specific effector T cells (Dangles *et al*, 2002, 2003; Ghosh *et al*, 2005a). We intended to verify if similar events occur when melanoma cells are cultured in MCTS and the eventually underlying molecular mechanisms.

Percentages of TAA-specific T cells ‘*ex vivo*’ are extremely low, and usually do not exceed 0.5% of total CD8+ T cells, at best (Seiter *et al*, 2002). Thus, to perform our studies in controlled conditions, we again resorted to the use of antigen-specific cloned T cells as effectors. Importantly, CTL clones are far more efficient in this respect than T cells freshly obtained from peripheral blood (Pittet *et al*, 2003). As tumour cells cultured in 3D are unfit to serve as targets for ^51^Cr release assays, because washing steps required after labelling disrupt cell aggregates, we turned to IFN-*γ* production by antigen-specific T cells following interaction with targets expressing appropriate TAA and restriction determinants, as a classical alternative antigen recognition assay (Butterfield *et al*, 1999; Pelfrey *et al*, 2000).

HBL and D10 HLA-A^*^0201+ melanoma cells expressing Melan-A/MART-1 and gp100 differentiation TAA cultured in 2D or 3D were used to stimulate IFN-*γ* production by previously characterised specific CTL clones (Zajac *et al*, 2003). NA8 cells (HLA-A^*^0201+, TAA−) were used as negative controls.

**Figure 3:**
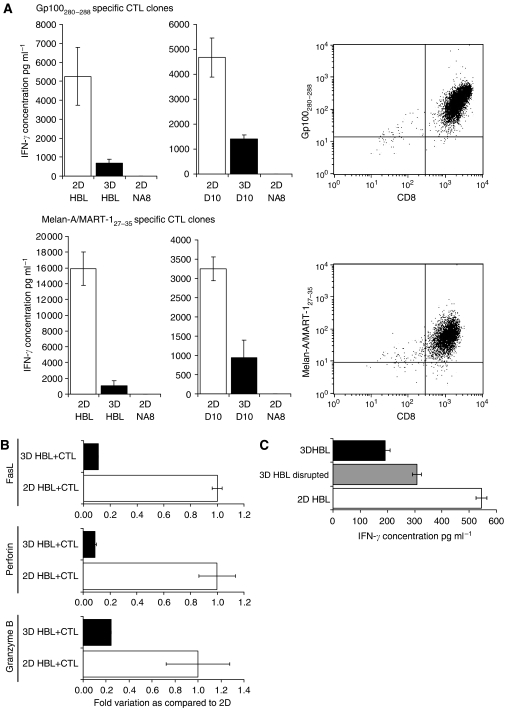
Functions of TAA-specific CTL are impaired when target melanoma cells are cultured in MCTS. (**A**) Interferon-gamma secretion by CTL clones upon stimulation with HBL, D10, or NA8 cells cultured in 2D or in MCTS. Cytotoxic T-lymphocyte clones specific for HLA-A^*^0201-restricted gp100_280–288_ or Melan-A/MART-1_27–35_ epitopes and displaying corresponding tetramer binding profiles (right panels) were co-incubated for 24 h at 2.5 : 1 E:T ratio in the presence of similar numbers of HBL or D10 melanoma cells (HLA-A^*^0201+,gp100+,Melan-A/MART-1+), cultured in 2D (□) or in 3D (▪). Interferon-gamma secretion was measured by ELISA in culture supernatants. Data are reported as average of triplicate measurements. Error bars represent the standard deviation of the mean concentration of triplicate cultures. (**B**) The FasL, perforin, and granzyme B gene expression in CTL stimulated by 2D or 3D cultured HBL. Cells from a CTL clone specific for HLA-A^*^0201-restricted Melan-A/MART-1_27–35_ epitope were co-cultured for 24 h at 2.5 : 1 E:T ratio in the presence of HBL melanoma cells cultured in 2D (□) or in 3D (▪). Total cellular RNA was extracted and reverse transcribed. FasL, perforin, and granzyme B gene expression were analysed by quantitative real-time PCR. Data were expressed as ratio to cells incubated in the presence of melanoma cells cultured in 2D. (**C**) Interferon-gamma secretion by CTL cultured with HBL cells from intact or disrupted MCTS. Cells from a CTL clone specific for HLA-A^*^0201-restricted Melan-A/MART-1_27–35_ epitope were stimulated for 24 h at 1 : 1 E:T ratio in the presence of HBL melanoma cells cultured in 2D (□), in 3D (▪) or following MCTS disruption (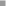). Interferon-gamma secretion was measured by ELISA in culture supernatants. Data are reported as average of triplicate measurements. Error bars represent the standard deviation of the mean concentration of triplicate cultures.

As expected, HBL and D10 cells cultured in 2D induced high IFN-*γ* production in gp100_280–288_ or Melan-A/MART-1_27–35_-specific CTL clones. In sharp contrast, considerably lower amounts of cytokine were produced if CTL were stimulated with spheroids of 30 000 HBL or D10 target cells following 3-day culture in 3D. Similar results were obtained in independent experiments performed by using different gp100_280–288_ or Melan-A/MART-1_27–35_ specific, HLA-A^*^0201 restricted CTL clones, at different E : T ratios. Representative data are reported in Figure 3A.

These differences tended to disappear when CTL were challenged with spheroids containing ⩽2000 cells (not shown). These data suggest that polyHEMA ‘*per se*’ is not toxic to CTL.

Elicitation of CTL functions relies on the expression of an array of components of their lytic machinery. For instance, CTL may kill through Fas pathway (Ostergaard *et al*, 1987; Rouvier *et al*, 1993), and granzymes entering target cells (Froelich *et al*, 1996; Jans *et al*, 1998; Pinkoski *et al*, 1998; Edwards *et al*, 1999) may rapidly induce their DNA fragmentation-based apoptosis (Heusel *et al*, 1994; Shresta *et al*, 1995). Furthermore, perforin in the CTL granules plays a pivotal role in granule-mediated killing (Helgason *et al*, 1992; Kagi *et al*, 1994; Kojima *et al*, 1994; Lowin *et al*, 1994). Thus, we also assessed CTL functions by evaluating FasL, granzyme B, and perforin gene expression in CTL co-cultured with melanoma target cells growing in 2D or in MCTS (Figure 3B). As expected, interaction with targets Melan-A/MART-1_27–35_+HLA-A^*^0201+ cultured in 2D resulted in the expression of FasL, granzyme B, and perforin genes in antigen-specific CTL. However, the expression of these genes was significantly lower when effector cells were stimulated by MCTS.

These data indicated that antigen recognition by CTL can be impaired if target cells are cultured in 3D, rather than in monolayers. A possible explanation for this observation could be offered by mere structural considerations: culture in spheroids may provide an overall smaller cell surface accessible to CTL attack, as compared to monolayers, resulting in decreased activation of effector cells. To address this issue, HBL cells were cultured in MCTS and subsequently disaggregated. The resulting cell suspensions were used to stimulate antigen-specific CTL. Melanoma cells from freshly disrupted spheroids indeed induced IFN-*γ* secretion in Melan-A/MART-1_27–35_-specific CTL clones to levels intermediate between those induced by 2D or 3D cultured HBL (Figure 3C). These results indicate that the observed impairment of antigen recognition by CTL is at least in part due to the smaller cell surface accessible to effectors, but it cannot be exclusively ascribed to structural hindrances.

### Melan-A/MART-1, gp100 and tyrosinase expression in HBL and D10 cells cultured in 2D and 3D

**Figure 4:**
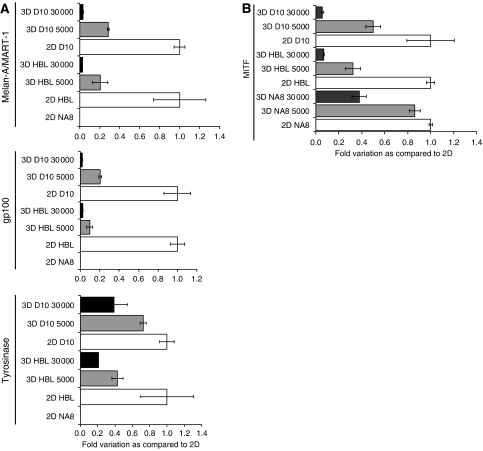
Tumour-associated antigens expression in melanoma cells cultured in 2D or in MCTS. (**A**) Melan-A/MART-1, gp100, and tyrosinase gene expression in melanoma cells cultured in 2D or in MCTS at different cell densities. HBL and D10 cells were cultured for 3 days in monolayer or in 3D at different cell densities (5000 and 30 000 cells). Total cellular RNA was extracted, reverse transcribed. Melan-A/MART-1, gp100, and Tyrosinase gene expression were analysed by quantitative real-time PCR. Data were expressed as ratio to the corresponding 2D sample. NA8 cells, known to be negative for TAA expression, were used as negative control. (**B**) Microphtalmia-associated transcription factor gene expression in melanoma cells cultured in 2D or in MCTS at different cell densities. Melanoma cells (HBL, D10 and NA8) were cultured for 3 days in 2D (□) or in 3D (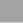: 5000 cells – ▪: 30 000 cells). Gene expression was analysed by quantitative real-time PCR. Data are expressed by using, as reference, specific gene expression observed in the corresponding 2D sample.

Previous work suggests that high cell density in monolayer cultures could affect TAA expression (Ramirez-Montagut *et al*, 2000). In an attempt to clarify molecular mechanisms underlying our observations, we addressed antigen expression in target cells. Thus, we comparatively explored the expression not only of Melan-A/MART-1 and gp100 but also of tyrosinase genes, encoding differentiation antigens widely used in active specific immunotherapy (Zajac *et al*, 2003) in HBL and D10 melanoma cells cultured in either MCTS or conventional 2D conditions. NA8 cells (Certa *et al*, 2001) were used as negative controls (Figure 4A).

Quantitative real-time PCR analysis revealed that HBL and D10 cells cultured in MCTS display a significantly lower (⩽40%) expression of Melan-A/MART-1, gp100 and tyrosinase differentiation TAA as compared to similar numbers of cells cultured in 2D conditions. Similar profiles were obtained using 18S as house-keeping gene. Importantly, this downmodulation was visible in 3-day-old spheroids, well before the appearance of inner necrotic cores on days 10–12 (Walenta *et al*, 1990; Santini and Rainaldi, 1999; Ghosh *et al*, 2005b). Most interestingly, it was clearly related to cell density. Indeed, the highest downregulation of the expression of genes encoding differentiation TAA was detectable in spheroids containing 30 000 melanoma cells, whereas milder effects were recorded in 3D structures including 5000 tumour cells.

Interestingly, HBL and D10 cells from aggregates disrupted by vigorous pipetting and subsequently cultured in monolayers could not recover baseline (2D) TAA gene expression before 3–5 days (data not shown).

As hypoxia may represent an early event in inner MCTS layers, leading, as reviewed previously (Douglas and Haddad, 2003; Zhou *et al*, 2006), to typical gene expression profiles, we investigated whether it could play a role in the downregulation of the expression of melanoma differentiation antigens observed in spheroids. Monolayer cultures of D10 and HBL cell lines were incubated in low (5%) pO_2_ for 3 days and the expression of the genes under investigation was subsequently assessed. In no case we observed a significant downmodulation of melanoma TAA in cells cultured in hypoxic conditions (data not shown).

Taken together, these data indicated that the architecture of cultures might regulate the expression of melanoma differentiation antigens.

It has been recently reported that OSM, produced by melanoma cells actively downregulates Melan-A/MART-1 mRNA transcription inducing antigen silencing in tumour cells (Durda *et al*, 2003). On the other hand, Melan-A/MART-1 and gp100 gene expression have been shown to be transcriptionally controlled by MITF (Du *et al*, 2003), the master regulator of melanocytic differentiation.

We explored comparatively the expression of the genes encoding these soluble factors potentially involved in the modulation of antigen expression in HBL, D10, and NA8 melanoma cells cultured in either MCTS or conventional 2D conditions (Figure 4B). While OSM gene expression was not detectable (data not shown), MITF gene expression was downregulated in the three cell lines under investigation, when cultured in MCTS as compared to 2D. These effects appeared to be cell density dependent. Furthermore, they did not result from an overall decrease of metabolic activities of cell cultured in 3D. Indeed, our own oligonucleotide chip hybridisation experiments (Ghosh *et al*, 2005b) clearly indicated that upregulation of a number of genes takes place in the melanoma cells under investigation when cultured in 3D.

### Modulation of HLA class I expression in spheroids of different sizes

**Figure 5:**
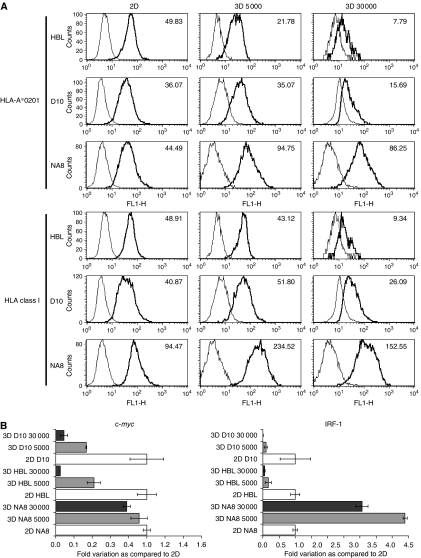
HLA class I expression in melanoma cells cultured in 2D or in MCTS. (**A**) Flow cytometric analysis of HLA-A^*^0201 and HLA Class I expression in melanoma cells cultured in 2D or in MCTS at different cell densities. The HBL, D10, and NA8 cells were cultured for 7 days in 2D or 3D at the indicated cell densities. Cells were then harvested, and aggregates were disrupted by vigorous pipetting and trypsinisation. Cells were stained either with control antibodies (thin lines) or with mAbs recognising HLA-A^*^0201 or a monomorphic epitope on HLA class I heavy chains (bold lines). Relevant mean fluorescence intensities, reported in individual histograms, were calculated by subtracting values deriving from isotype control staining from experimental values. (**B**) Expression of c-*myc* and IRF-1 genes in melanoma cells cultured in 2D or in MCTS at different cell densities. HBL, D10, or NA8 cells were cultured for 3 days in 2D (□) or in 3D (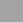: 5000 cells – ▪: 30 000 cells). Gene expression was analysed by quantitative real-time PCR and results are expressed as ratio to specific gene expression, as observed in the corresponding 2D sample used as reference.

The expression of HLA class I molecules and, in particular, of HLA-A^*^0201, the allele restricting the immunodominant CTL response to the gp100 and Melan-A/MART-1 epitopes under investigation was also evaluated in melanoma cells cultured in MCTS as compared to 2D. Figure 5A reports data from one representative experiment out of three performed. HBL MCTS (30 000 cells per spheroids) displayed a marked (>fivefold) decrease in HLA-A^*^0201 expression at the protein level, as compared to 2D cultures. This downmodulation was not allele specific, as similar results were obtained by using a mAb specific for a monomorphic epitope on HLA class I heavy chain. This reduction also appeared to be cell density dependent. In D10 MCTS a significant (⩾twofold) decrease in HLA molecules expression, as compared to 2D cultures, was also observed.

Notably, however, this was not a constant finding in all the cell lines under investigation. For instance, NA8 melanoma cells (HLA-A^*^0201+, TAA−) displayed a divergent HLA modulation pattern as compared to HBL and D10. Indeed, when cultured in MCTS, NA8 showed significant (⩾twofold) increases in HLA-A^*^0201 and overall HLA class I expression, especially at a cell density of 5000 cells per spheroids.

HLA class I gene expression is regulated by transcription factors of the IRF family (Girdlestone *et al*, 1993), whereas c-*myc* has been shown to downregulate HLA class I expression in human melanoma (Versteeg *et al*, 1988).

Consistent with the HLA expression data observed at the protein level, IRF-1 gene expression was also cell density dependently downregulated in HBL and D10 cultured in MCTS as compared to 2D. Accordingly, IRF-1 gene expression was upregulated in NA8 MCTS in comparison with cells cultured in monolayers.

On the other hand, surprisingly, c-*myc* expression was significantly downregulated in D10 and HBL spheroids, as compared to cells cultured in monolayer, but it was unaffected in NA8 spheroids (Figure 5B).

### Role of lactic acid in the defective recognition by TAA-specific CTL of tumour cells cultured in spheroid

Cancer cells are known to be characterised by high production of lactic acid under aerobic conditions (Warburg, 1956; Kim and Dang, 2006). Recently, it has been shown that production of lactic acid is enhanced in tumour cells cultured in spheroids, as compared to monolayers (Gottfried *et al*, 2005). Most importantly, lactic acid, at the concentrations produced by tumour cells in these culture conditions inhibits the proliferation of antigen specific CTL lines co-cultured with autologous dendritic cells in the presence of antigenic peptides (Gottfried *et al*, 2005). Prompted by this report, we evaluated the production of lactic acid by the melanoma cell lines under investigation and its eventual role in the defective antigen recognition by specific CTL.

**Figure 6:**
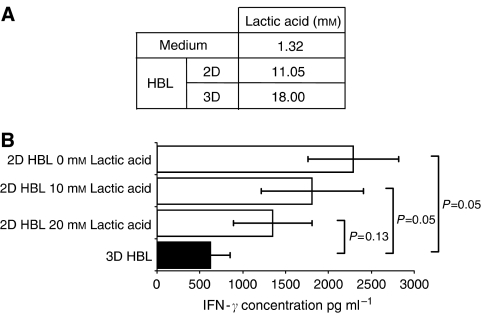
Lactic acid production by tumour cells cultured in different conditions and its role in antigen recognition by CTL. (**A**) Lactic acid production by melanoma cells cultured in 2D or in 3D. The HBL cells were cultured in standard monolayers or in MCTS (30 000 cells) for 3 days. Supernatants were then harvested and their lactic acid content was measured as described in ‘Materials and Methods’. (**B**) Effects of lactic acid on antigen recognition by CTL. Cytotoxic T-lymphocyte from a MelanA/MART-1_27–35_-specific HLA-A^*^0201 restricted clone were co-incubated with HBL melanoma cells cultured in 2D (□) or 3D (▪), as indicated, in the presence of lactic acid at the indicated concentrations.

A 3-day culture of HBL cells in MCTS induced a >60% increase in their lactic acid production (Figure 6A), as compared to monolayer cultures. We then performed our antigen recognition assays by using as targets HBL melanoma cells cultured in 2D in the absence or in the presence of graded concentrations of exogenous lactic acid (Sigma-Aldrich, St Louis, MO, USA).

As expected, target cells cultured in 2D-induced antigen-stimulated IFN-*γ* production by a Melan-a/MART-1_27–35_ HLA-A^*^0201-restricted CTL clone to an extent significantly higher than cells cultured in MCTS. However, addition of exogenous lactic acid to target cell monolayers resulted in a trend towards decreasing IFN-*γ* production. In particular, in the presence of 20 mM lactic acid, target cell monolayers induced CTL responses not significantly different from those triggered by target cells cultured in MCTS in the absence of exogenous lactic acid (Figure 6B).

## DISCUSSION

The past decade has witnessed an unprecedented wave of cancer immunotherapy trials, prompted by the identification of large numbers of TAA and by major advances in basic immunology. Most of these efforts have targeted metastatic melanoma. A large majority of published reports suggest that a variety of different vaccination procedures are capable of inducing TAA-specific CTL in high percentages of immunised patients. However, clinical responses are detectable only in a minority of them. These data underline that even in the presence of specific immune response, tumours may be relatively insensitive to its effects.

Molecular mechanisms underlying the discrepancy between immunological and clinical responsiveness to active antigen-specific immunotherapy have been investigated by several groups.

Tumour escape from CTL recognition has been attributed to downregulation of TAA or HLA class I molecules expression (Marincola *et al*, 2000) possibly resulting from the selection of resistant variants in neoplastic cell populations exposed to immunological pressure. However, this mechanism, whose *in vivo* relevance is debated, might indirectly support the concept of a clinical efficacy of CTL induction, whose evidence is mostly missing (Marincola *et al*, 2003).

More recently, the discrepancy between induction of TAA-specific immune responses and clinical responsiveness has also been attributed to CTL defects. TAA-specific T cells sampled *ex vivo* from tumour metastases have been shown to be quiescent (Monsurro *et al*, 2003, 2004; Zippelius *et al*, 2004), and characterised by an impaired capacity to produce IFN-*γ* upon antigenic challenge. Still unclear, however, is the role of the tumour cells, if any, in inducing such state.

These reports urge the development of novel *in vitro* models utilising human cells and permitting controlled investigations of the interaction between tumour cells and the immune system.

Data from different groups, including ours, indicate that culture of tumour cells in 3D structures modulates their gene expression profiles and decreases their susceptibility to the immune-mediated CTL attack, although still unclear are the underlying molecular mechanisms (Ghosh *et al*, 2005a, 2005b).

Here, we show that TAA-specific CTL are unable to penetrate in deep the 3D architecture of MCTS, closely reminding the ‘non-brisk’ infiltration of melanoma by T cells observed in clinical cancer tissues (Mihm *et al*, 1996; Bernsen *et al*, 2004). This lack of infiltration suggested a possible defective killing of tumour cells cultured in spheroids. Indeed, our study shows that lower amounts of IFN-*γ* are produced by CTL stimulated by melanoma cells cultured in MCTS as compared to those cultured with tumour cell monolayers. The concept of impaired elicitation of CTL functions was reinforced by decreases in FasL, granzyme B, and perforin gene expression in antigen-specific CTL when cultured with MCTS as compared with their counterparts cultured in 2D.

Our data indicate that a multiplicity of mechanisms concur in decreasing the susceptibility of melanoma cells cultured in MCTS to the effects of antigen-specific CTL, as compared with cell monolayers.

First, 3D structures *per se*, limit the capacity of effector cells in recognising HLA class I restricted antigens possibly by merely reducing the cell surface exposed to CTL. This mechanism, however, is only partially responsible for the impaired antigen recognition, as CTL cultured with melanoma cells from disrupted MCTS secreted IFN-*γ* at a level intermediate between 2D and MCTS.

Second, the expression of melanoma differentiation antigens is downregulated in tumour cells cultured in 3D as compared with monolayers. In our hands, this is neither related to hypoxia nor to increased OSM gene expression but rather to a decreased MITF gene expression and to the high cell concentrations elicited by culture in MCTS.

Third, the surface expression of HLA class I molecules can be downregulated in melanoma cells cultured in 3D, as compared with their counterparts in 2D.

These features have been detected relatively frequently in clinical melanoma specimen. Their occurrence has been attributed to the outgrowth of cancer cells characterised by low expression of TAA and/or restricting HLA class I determinants following exposure of tumours to immunoselective pressures (Marincola *et al*, 2000). However, our data suggest that a low expression of HLA class I molecules and at least of melanoma differentiation antigens in tumours, could be inherent in their 3D growth, even in the absence of an exogenous immune pressure.

Fourth and finally, lactic acid production by melanoma cells is increased if they are cultured in MCTS, as compared with monolayer cultures, and lactic acid significantly inhibits TAA-triggered IFN-*γ* production by specific CTL. Consistent with previous reports (Gottfried *et al*, 2006), these effects appear to be mediated by functional inhibition of effector cells, as no downregulation of TAA expression is detectable in melanoma cells cultured in these conditions. Interestingly, lactic acid produced within melanoma and prostate cancer MCTS has also been shown to impair the phenotypic and functional maturation of infiltrating dendritic cells thereby inhibiting their antigen-presenting capacity (Gottfried *et al*, 2006).

Most importantly, none of these mechanisms alone is able to entirely account for the inhibition of antigen recognition by specific CTL, detectable upon culture in the presence of melanoma cells cultured in 3D, as opposed to 2D. Their combination, however, elicits powerful inhibitory effects.

We are fully aware that culture of melanoma cell lines in 3D might only partially reflect the complexity of solid tumours developing *in vivo*. However, the clear discrepancy between data obtained by applying techniques of current use for the *in vitro* detection of antitumour responses and clinical evidence urges the development of alternative experimental models. Multicellular tumour spheroids may then qualify as technology of choice for the screening not only of novel drugs, but also of immune-mediated therapeutic procedures.

Further research is warranted to explore in this controlled *in vitro* model the consequences on effector cells of their interaction with tumour cells growing in 3D architectures, as opposed to growing in monolayers or in suspension.

Importantly, providing a molecular background to widespread clinical experience, our data suggest that the effectiveness of antitumour immune response may largely depend not only on affinity and functional capacities of effector cells, but also on the structural characteristics of the growth of cancer cells, rather than on their mere numbers, and strongly support the use of active antigen-specific immunotherapies in minimal residual disease states.
